# The RS504393 Influences the Level of Nociceptive Factors and Enhances Opioid Analgesic Potency in Neuropathic Rats

**DOI:** 10.1007/s11481-017-9729-6

**Published:** 2017-03-23

**Authors:** Klaudia Kwiatkowski, Anna Piotrowska, Ewelina Rojewska, Wioletta Makuch, Joanna Mika

**Affiliations:** Department of Pain Pharmacology, Institute of Pharmacology Polish Academy of Sciences, 12 Smetna Str., 31-343 Krakow, Poland

**Keywords:** Buprenorphine, CCR2, Interleukins, Morphine, RS504393

## Abstract

Increasing evidence has indicated that activated glial cells releasing nociceptive factors, such as interleukins and chemokines, are of key importance for neuropathic pain. Significant changes in the production of nociceptive factors are associated with the low effectiveness of opioids in neuropathic pain. Recently, it has been suggested that CCL2/CCR2 signaling is important for nociception. Here, we studied the time course changes in the mRNA/protein level of CD40/Iba-1, CCL2 and CCR2 in the spinal cord/dorsal root ganglia (DRG) in rats following chronic constriction injury (CCI) of the sciatic nerve. Moreover, we examined the influence of intrathecal preemptive and repeated (daily for 7 days) administration of RS504393, CCR2 antagonist, on pain-related behavior and the associated biochemical changes of some nociceptive factors as well as its influence on opioid effectiveness. We observed simultaneous upregulation of Iba-1, CCL2, CCR2 in the spinal cord on 7th day after CCI. Additionally, we demonstrated that repeated administration of RS504393 not only attenuated tactile/thermal hypersensitivity but also enhanced the analgesic properties of morphine and buprenorphine under neuropathy. Our results proof that repeated administration of RS504393 reduced the mRNA and/or protein levels of pronociceptive factors, such as IL-1beta, IL-18, IL-6 and inducible nitric oxide synthase (iNOS), and some of their receptors in the spinal cord and/or DRG. Furthermore, RS504393 elevated the spinal protein level of antinociceptive IL-1alpha and IL-18 binding protein. Our data provide new evidence that CCR2 is a promising target for diminishing neuropathic pain and enhancing the opioid analgesic effects.

## Introduction

The treatment of neuropathic pain is a serious clinical problem, mainly because of poor responses and numerous undesired adverse effects of opioids that are commonly used in high doses. Pain associated with neuropathy develops as a result of central and/or peripheral nervous system damage; however, the detailed mechanism of its development remains unclear. Undoubtedly, alongside neuronal pathways, activated glial cells that produce nociceptive factors, such as interleukins and chemokines, are of key importance for pain transmission under neuropathy (Gao and Ji 2010; Kiguchi et al. 2012; Old et al. 2015). Until now, it has been well established that the disrupted equilibrium between pro- and antinociceptive factors contributes to the development of neuropathic pain (Rojewska et al. 2014b; Pilat et al. 2015, 2016).

Several studies have shown that intrathecal/intracerebroventricular injections of IL-1beta (Oka et al. 1994; Mika et al. 2008) and IL-6 (Oka et al. 1995) induced pain-related behavior, whereas intrathecal administration of IL-1alpha (Mika et al. 2008), IL-1 receptor antagonist (IL-1RA) (Milligan et al. 2006; Ledeboer et al. 2007; Pilat et al. 2015) and IL-18 binding protein (IL-18BP) (Pilat et al. 2016) attenuated neuropathic pain in rats. Moreover, it was recently reported that intrathecal IL-10 gene therapy resulted in profound neuropathic pain relief in several animal models (Milligan et al. 2006; Ledeboer et al. 2007). Previous studies have also shown that intracerebroventricular injections of exogenous IL-1beta reduced the analgesic effect of morphine (Gul et al. 2000; Rady and Fujimoto 2001), whereas chronic administration of IL-1RA (Pilat et al. 2015), IL-18BP (Pilat et al. 2016), sTNFR, and anti-IL-6 antibody (Raghavendra et al. 2002) partially restored morphine efficacy in neuropathic rats. Additionally, a single intrathecal injection of IL-1RA and IL-18BP improved the analgesic efficiency of both morphine and buprenorphine in sciatic nerve-injured rats (Pilat et al. 2015, 2016).

Both interleukins and chemokines are involved in pain processing (Gao and Ji 2010; Old and Malcangio 2012; Kwiatkowski et al. 2016). It has already been shown that C-C motif chemokine ligand 2 (CCL2; *Monocyte Chemoattractant Protein-1*; MCP-1) and its receptor C-C chemokine receptor type 2 (CCR2) play crucial roles in neuropathic pain development (Gao and Ji 2010; Van Steenwinckel et al. 2011; Zhang et al. 2012). Piotrowska et al. (2016a) provided evidence that RS504393 decreased pain-related behavior in rats following chronic constriction injury by reducing microglial activation and spinal upregulation of CCL2 and CCR2.

The first aim of our study was to examine time course changes in the mRNA and protein level of CD40/Iba-1, CCL2 and CCR2 in the spinal cord and dorsal root ganglia (DRG) in rats following chronic constriction injury (CCI) of the sciatic nerve. The second object of our study was to examine how the RS504393, CCR2 antagonist, influences tactile and thermal hypersensitivity in CCI-exposed rats. The next goal was to examine the impact of the repeated intrathecal administration of the RS504393 on the protein level of Iba-1, CD4 and CD8, as well as mRNA and protein levels of pronociceptive (IL-1beta, IL-18, IL-6, and iNOS) and antinociceptive (IL-1alpha, IL-1RA, IL-18BP, and IL-10) factors in the spinal cord and DRG during neuropathic pain, thereby to enhance our knowledge regarding its analgesic mechanism of action. The final aim of our study was to examine how and if RS504393 influences the analgesic effects of morphine and buprenorphine in rats following chronic constriction injury to the sciatic nerve.

## Materials and Methods

### Animals and Ethical Statement

Adult male Wistar rats (250–300 g) from Charles River Laboratories International, Inc. (Germany) were used in our experiments. Rats were housed in cages lined with sawdust under a standard 12/12 h light/dark cycle (lights on at 8.00 a.m.) with food and water available *ad libitum*. Animals were allowed to acclimate to the environment for approximately 5 min prior to behavioral testing. All experiments were performed according to the recommendations of the International Association for the Study of Pain (IASP) (Zimmermann 1983) and the National Institutes of Health (NIH) Guide for the Care and Use of Laboratory Animals. The study protocol was approved by the II Local Bioethics Committee branch of the National Ethics Committee for Experiments on Animals based at the Institute of Pharmacology, Polish Academy of Sciences (Krakow, Poland).

### Intrathecal Implantation of Catheters

Catheters for intrathecal (*i.t.*) injections were implanted according to the method described by Yaksh and Rudy (1976) and our earlier publications (Popiolek-Barczyk et al. 2015; Rojewska et al. 2015; Piotrowska et al. 2016a). Just before operation each rat was anesthetized with sodium pentobarbital (60 mg/kg) administered intraperitoneally (*i.p.)*. The *i.t.* catheter consisted of 13 cm-long polyethylene tubing (PE 10, Intramedic; Clay Adams, Parsippany, NJ, USA) with the dead space of 10 μl sterilized by immersion in 70% (*v*/v) ethanol and fully flushed with *aqua pro injectione* before insertion. Then, 7.8 cm of catheter was carefully introduced through the atlanto-occipital membrane and advanced into the subarachnoid space at the rostral level of the spinal cord lumbar enlargement (L4-L5). The first injection of water (10 μl) was slowly performed after implantation, and the catheter was tightened. All rats were allowed to recover from surgery for one week before creating the neuropathic pain model.

### Chronic Constriction Injury of the Sciatic Nerve in Rats

Chronic constriction injury (CCI) of the sciatic nerve was performed under sodium pentobarbital anesthesia (60 mg/kg, *i.p.*) according to the procedure described by Bennett and Xie (1988). First, an incision below the hipbone was made, and then the *biceps femoris* and *gluteus superficialis* were separated. After exposing the right sciatic nerve, four ligatures (4/0 silk) were loosely tied around the nerve at 1-mm spacing until a brief twitch in the operated hind limb was elicited. All rats developed long-lasting neuropathic pain. In previous research, we showed that there is no significant difference in the nociceptive responses as well as the level of nociceptive factors between naive and sham-operated animals (e.g., IL-18, IL-6, and IL-1 beta protein levels were 1 ± 0.2 vs. 0.9 ± 0.2, 1 ± 0.1 vs. 1.1 ± 0.1, 1 ± 0.1 vs. 1.12 ± 0.1, respectively), and therefore we used naive animals for the behavioral experiments in the current study (Rojewska et al. 2015).

### Behavioral Tests

#### Tactile Hypersensitivity Measurement

Tactile hypersensitivity in the rats was measured using an automatic von Frey apparatus (Dynamic Plantar Aesthesiometer Cat. No. 37400, Ugo Basile, Italy). The animals were placed in plastic cages with a wire net floor 5 min before the experiment. Von Frey filaments were applied in increasing values (up to 26 g) to the midplantar surface of the hind paw, and measurements were taken automatically, as described previously (Mika et al. 2010; Makuch et al. 2013). The ipsilateral paw was tested two times in 2 min intervals, and the mean value was calculated.

#### Thermal Hypersensitivity Measurement

Thermal hypersensitivity was assessed using a cold plate apparatus (Cold/Hot Plate Analgesia Meter No. 05044, Columbus Instruments, USA). The animals were placed on the cold plate, and the latency to lift the hind paw was recorded. The temperature of the plate was kept at 5 °C, and the cut-off latency was 30 s. In all cases, the injured paw reacted first (Mika et al. 2007; Makuch et al. 2013).

### Drug Administration

**Scheme 1:**
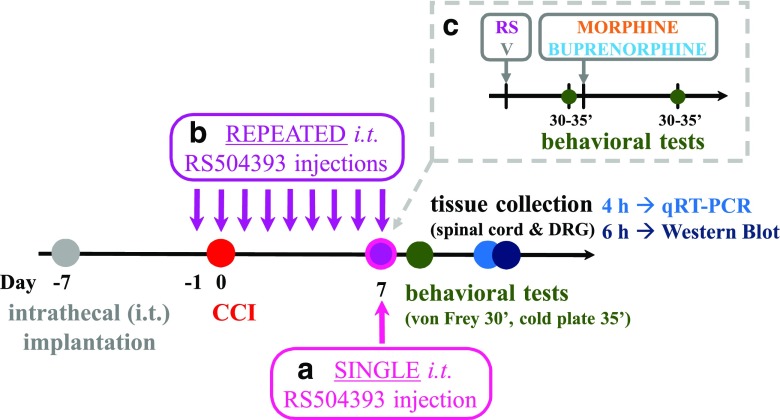
All behavioral experiments were preceded by intrathecal implantation of catheters since all drugs were administered via intrathecal (*i.t.*) injection. One week after implantation, all rats were subjected to sciatic nerve chronic constriction injury (CCI; day 0). **a** The single injection of RS504393 (20 μg/5 μl) or vehicle was performed 7 days after CCI after which behavioral tests were conducted 30 min (von Frey) and 35 min (cold plate) (data presented on Fig. 2a and c). **b** The repeated injection of RS504393 (20 μg/5 μl) or vehicle were provided once a day 16 h (day −1) and 1 h (day 0) before CCI and for 7 next days. On the 7th day post-CCI behavioral tests were performed 30 min (von Frey) and 35 min (cold plate) after drug administration and then 4 h (for RT-qPCR analysis) or 6 h (for Western blot analysis) after the last RS/V injection the tissue was collected (data presented on Fig. 2b and d). **c** Additionally, the coadministration of repeated RS504393 with opioids were performed. The group of chronically RS- or V-treated rats received on the 7th day after CCI (approximately 60 min after RS/V injection) a single injection of morphine or buprenorphine (2.5 μg/5 μl), and then, the von Frey test (30 min) and the cold plate test (35 min) were performed (data presented on Fig. 7a and b). *Abbreviations: CCI, chronic constriction injury; DRG, dorsal root ganglia; i.t., intrathecal; RT-qPCR, quantitative reverse transcriptase real-time polymerase chain reaction; RS, RS504393; WB, Western blot; V, vehicle*

Following substances were used in this study: RS504393 (**RS**; Tocris, Warsaw, Poland), morphine hydrochloride (**M**; TEVA, Kutno, Poland), and buprenorphine (**B**; Polfa Warszawa S.A., Warsaw, Poland). RS (20 μg/5 μl) was dissolved in 12% DMSO and intrathecally administered preemptively at 16 h and 1 h before CCI and then once a day for 7 days or only once on the 7th day post-CCI. The control group received vehicle (**V**; 12% DMSO) at the same schedule. The concentration of examined substances were selected according to the literature (Zhu et al. 2014; Piotrowska et al. 2016a) and our preliminary study. The dosing schedule (Scheme 1) was selected basing on our previous research, which demonstrated that substances which may modulate microglial cells need to be administered preemptively (Rojewska et al. 2015; Kwiatkowski et al. 2016; Jurga et al. 2016) as the activated microglia is very difficult to fade out afterwards. Respective substances were slowly delivered in a volume of 5 μl via the *i.t.* catheter and followed by 10 μl of *aqua pro injectione*. The behavioral tests were performed 7 days after CCI always in the same order, firstly von Frey test and then cold plate test (similarly to our previous papers Mika et al. 2008; Makuch et al. 2013; Rojewska et al. 2014a; Kwiatkowski et al. 2016; Pilat et al. 2016; Rojewska et al. 2016; Zychowska et al. 2016), 30 min and 35 min after the last drug injection, respectively. On the 7th day post-CCI, chronically V-treated and RS-treated rats received a single dose of morphine or buprenorphine (2.5 μg/5 μl) 60 min after RS504393/vehicle injection, and then, both behavioral tests were repeated.

### Biochemical Tests

#### Analysis of Gene Expression (RT-qPCR)

Ipsilateral fragments of the dorsal part of the lumbar (L4-L6) spinal cord and the DRG (L4-L6) were collected immediately after decapitation, 4 h after the last injection of RS504393 on the 7th day post-CCI. Total RNA was extracted according to the method described by Chomczynski and Sacchi (Chomczynski and Sacchi 1987) using TRIzol reagent (Invitrogen). A NanoDrop ND-1000 spectrometer (NanoDrop Technologies, Wilmington, USA) was used to measure the RNA concentration in all probes. Reverse transcription was performed at 37 °C for 60 min using Omniscript reverse transcriptase (Qiagen Inc., Hilden, Germany) on 2 μg of total RNA from the tissue. The reaction was performed in the presence of an RNAse inhibitor (rRNasin, Promega, Mannheim, Germany) and oligo (dT16) primer (Qiagen Inc., Hilden, Germany). The resulting cDNA was diluted 1:10 with H_2_O, and approximately 50 ng of cDNA from each individual animal was used for each quantitative real-time PCR (RT-qPCR) reaction. The RT-qPCR was performed using Assay-On-Demand TaqMan probes, according to the manufacturer”s protocol (Applied Biosystems, Foster City, CA, USA), and run in an iCycler device (Bio-Rad, Hercules, Warsaw, Poland). A standard dilution curve determined the amplification efficiency for each assay. The following TaqMan primers and probes were used: Rn01527838_g1 (*HPRT*, hypoxanthine-guanine phosphoribosyltransferase); Rn01423590_m1 (*CD40*, cluster of differentiation 40); Rn00580555_m1 (*CCL2*, C-C motif chemokine ligand 2); Rn01637698_s1 (*CCR2*, C-C chemokine receptor type 2); Rn00566700_m1 (*IL-1alpha*, interleukin 1 alpha); Rn00580432_m1 (*IL-1beta*, interleukin 1 beta); Rn02586400_m1 (*IL-1RA*, interleukin 1 receptor antagonist); Rn00565482_m1 (*IL-1R1*, interleukin 1 receptor type 1); Rn01422083_m1 (*IL-18*, interleukin 18); Rn00584495_q1 (*IL-18BP*, interleukin 18 binding protein); RN01437151_m1 (*IL-18R*, interleukin 18 receptor); Rn00561420_m1 (*IL-6*, interleukin 6); Rn00563409_m1 (*IL-10*, interleukin 10); and Rn00561646_m1 (*iNOS*, inducible nitric oxide synthase). A standard dilution curve was used to determine the amplification efficiency for each assay (between 1.7 and 2). The cycle threshold values were calculated automatically by CFX Manager v.2.1 software according to the default parameters. RNA content was calculated as 2 - (threshold cycle). HPRT transcript level was not significantly changed in the CCI-exposed rats (Mika et al. 2010) and therefore, served as an adequate housekeeping gene.

#### Analysis of Protein Level (Western Blot)

Ipsilateral fragments of the dorsal part of the lumbar (L4-L6) spinal cord and the DRG (L4-L6) were collected immediately after decapitation, 6 h after the last injection of RS504393 on the 7th day post-CCI. First, tissue lysates were collected in RIPA buffer with a protease inhibitor cocktail, and then the reaction mixtures were cleared by centrifugation (14,000×g for 30 min at 4 °C). All samples (20 μg of protein) were heated in a loading buffer (4× Laemmli Buffer, Bio-Rad, Warsaw, Poland) for 5 min at 98 °C. Afterwards, all of them were resolved on 4–15% Criterion™ TGX™ pre-cast polyacrylamide gels (Bio-Rad, Warsaw, Poland) and transferred to Immune-Blot PVDF membranes (Bio-Rad, Warsaw, Poland) with semi-dry transfer (30 min, 25 V). Using 5% non-fat dry milk (Bio-Rad, Warsaw, Poland) in Tris-buffered saline with 0.1% Tween 20 (TBST), membranes were blocked for 1 h at RT, washed in TBST, and incubated overnight at 4 °C with the following primary antibodies: rabbit anti-Iba-1 (1:1000, Proteintech), rabbit anti-CCL2 (1:500, antibodies-online), rabbit anti-CCR2 (1:500, Abcam), mouse anti-CD4 (1:1000, R&D Systems), rabbit anti-CD8 (1:500, Santa Cruz), mouse anti-IL-1alpha (1:1000, Novus Biologicals), rabbit anti-IL-1beta (1:1000, Abcam), rabbit anti-IL-1RA (1:1000, Abcam), rabbit anti-IL-1R1 (1:1000, Sigma Aldrich), rabbit anti-IL-18 (1:1000, R&D Systems), rabbit anti-IL-18BP (1:1000, Novus Biologicals), rabbit anti-IL-18R (1:1000, Abcam), rabbit anti-IL-6 (1:500, Invitrogen), rabbit anti-IL-10 (1:500, Invitrogen), rabbit anti-iNOS (1:500, Santa Cruz) and mouse anti-GAPDH (1:5000, Millipore). Then, they were incubated for 1 h in horseradish peroxidase-conjugated anti-rabbit or anti-mouse secondary antibodies at a dilution of 1:5000. To dilute the primary and secondary antibodies, solutions from a SignalBoost™ Immunoreaction Enhancer Kit (Merck Millipore Darmstadt, Germany) were used. The membranes were washed 2 times for 2 min and 3 times for 5 min each with TBST. The final step was the detection of immunocomplexes conducted using the Clarity™ Western ECL Substrate (Bio-Rad, Warsaw, Poland) and visualized using a Fujifilm LAS-4000 FluorImager system. To quantify the relative levels of immunoreactivity, Fujifilm Multi Gauge software was used.

#### Statistical Analyses

The behavioral tests analyses (Figs. 2 and 7) are presented in grams and seconds as the mean ± standard error of the mean (SEM). One-way analysis of variance (ANOVA) was used to evaluate the experimental results. Differences between groups were analyzed with Bonferroni’s post-hoc test. ^***^ *p* < 0.001 indicates differences between naive rats and V-treated or RS-treated; ^###^ *p* < 0.001 indicates differences vs. V-treated CCI-exposed rats; ^&&&^ *p* < 0.001 indicates differences vs. RS-treated CCI-exposed rats; ^^^*p* < 0.001 indicates differences between V + M- or V + B-treated vs. RS + M- or RS + B-treated, CCI-exposed rats.

**Fig. 1:**
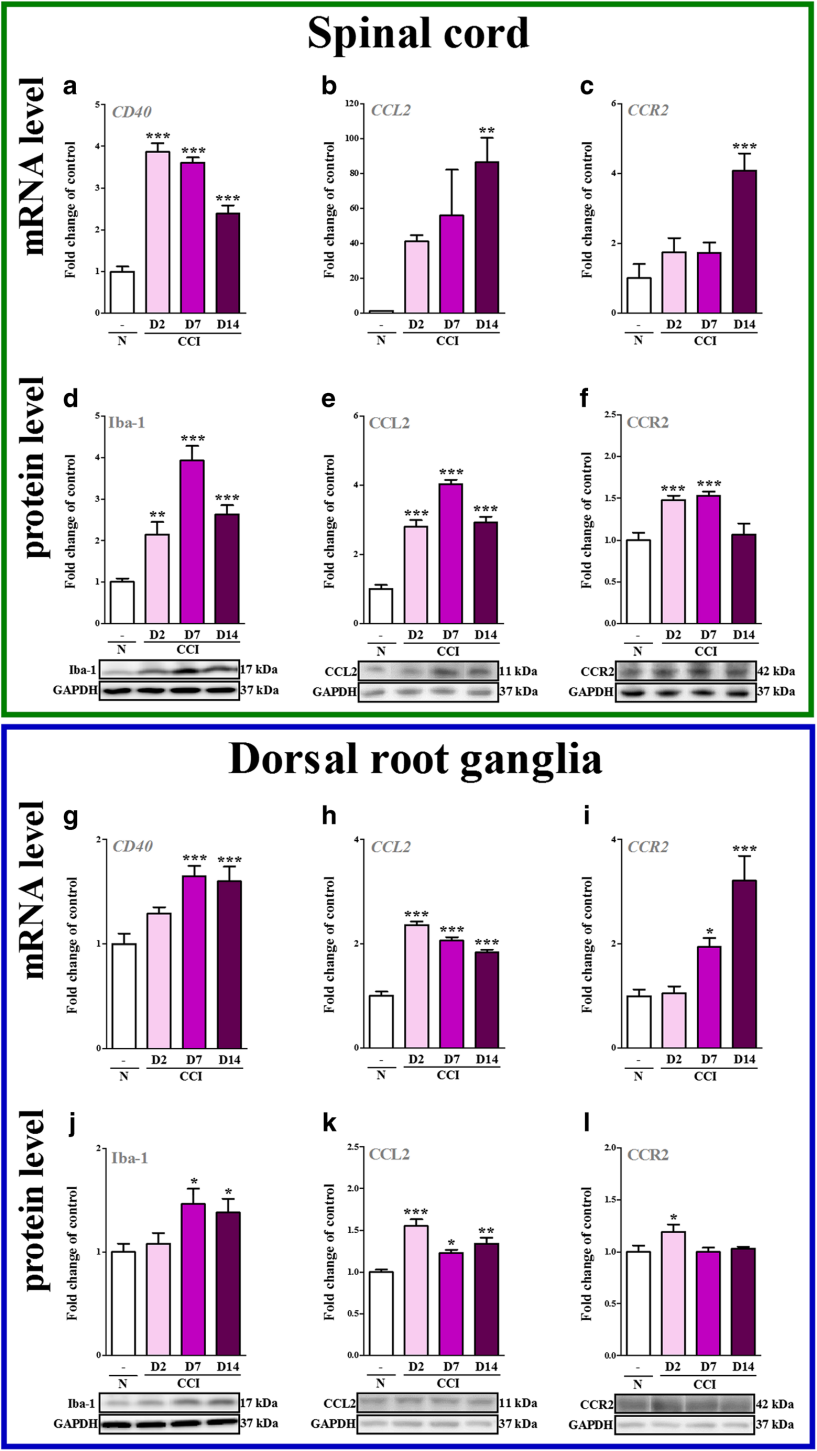
The time course changes in CD40, CCL2, CCR2 mRNA (**a**–**c**, **g**–**i**) and Iba-1, CCL2, CCR2 protein (**d**–**f**, **j**–**l**) levels in the spinal cord (**a**–**f**) and DRG (**g**–**l**) tissue on 2nd, 7th, and 14th days after chronic constriction injury (CCI) in rats. The data are presented as the mean ± SEM of 5–8 and 5–6 samples per group in RT-qPCR and Western blot analysis, respectively. The intergroup differences were analyzed using ANOVA with Bonferroni”s multiple comparisons test.﻿^*^ *p* < 0.05, ^**^ *p* < 0.01 and ^***^ *p* < 0.001 indicate differences between naive rats and CCI-exposed rats. *Abbreviations: CCI, chronic constriction injury; N, naive*

The biochemical data analyses (Figs. 1 and 3, 4, 5 and 6) are presented as the fold change compared to naive rats on the ipsilateral side of the dorsal lumbar spinal cord and DRG. The results were obtained from three groups of rats: naive, V-treated CCI-exposed and RS-treated CCI-exposed. The RT-qPCR and Western blot analysis data are presented as the mean ± SEM, which represents normalized averages. The intergroup differences were analyzed using ANOVA with Bonferroni”s multiple comparisons post-hoc test. ^*^ *p* < 0.05, ^**^ *p* < 0.01 and ^***^ *p* < 0.001 indicate differences between naive rats and V-treated or RS-treated CCI-exposed rats; ^#^ *p* < 0.05, ^##^ *p* < 0.01 and ^###^ *p* < 0.001 indicate differences vs. V-treated CCI-exposed rats.

All graphs and analyses were prepared using GraphPad Prism 5 (GraphPad Software, La Jolla, CA, USA).

## Results

### The Time Course Changes in CD40, CCL2, CCR2 mRNA and Iba-1, CCL2, CCR2 Protein Levels in the Spinal Cord and DRG as Measured on the 2nd, 7th and 14th Day after CCI

In the spinal cord the mRNA level of CD40 and protein level of Iba-1 had already been upregulated 3.9-fold (*p* < 0.001) and 2.15-fold (*p* < 0.01), respectively, 2 days after CCI (Fig. 1a and d). Strong changes (*p* < 0.001) in both CD40 and Iba-1 were still measured on 7th (3.6-fold and 3.9-fold, respectively) and 14th (2.4-fold and 2.6-fold, respectively) day post-CCI (Fig. 1a and d). The protein level of CCL2 in the spinal cord was significantly enhanced (2.8-fold, *p* < 0.001) on 2nd day (Fig. 1e). Even stronger upregulation of CCL2 protein level (4.0-fold, *p* < 0.001) was observed 7 days after CCI (Fig. 1e). On day 14th post-CCI we observed significant upregulation of both mRNA (86.3–fold, *p* < 0.01) and protein (2.9-fold, *p* < 0.001) level of CCL2 (Fig. 1b and e). The protein level of CCR2 at the spinal cord level had already been enhanced (1.5-fold, *p* < 0.001) 2 days after CCI (Fig. 1f). Similar changes (1.5-fold, *p* < 0.001) in CCR2 protein level were observed on 7th day after CCI (Fig. 1f). On day 14th post-CCI we observed significantly enhanced (4.1-fold, *p* < 0.001) CCR2 mRNA level (Fig. 1c) and no changes in protein level (Fig. 1f).

In the DRG no significant changes of both CD40 and Iba-1 were observed on 2nd day post-CCI (Fig. 1g and j). The upregulation of CD40 (1.29-fold, of the *p* < 0.001) and Iba-1 (1.46-fold, *p* < 0.05) was observed 7 days after CCI. Enhanced level of CD40 and Iba-1 lasted till 14th day (1.6-fold, *p* < 0.001 and 1.38-fold, *p* < 0.05, respectively) post-CCI (Fig. 1g and j). The mRNA and protein level of CCL2 in the DRG was strongly upregulated (2.35-fold, *p* < 0.001 and 1.55-fold, *p* < 0.001 respectively) on 2nd day and its enhanced expression slightly decrease on 7th (2.05-fold, *p* < 0.001 and 1.2-fold, *p* < 0.05, respectively) and 14th (1.8-fold, *p* < 0.001 and 1.3-fold, *p* < 0.01, respectively) day post-CCI (Fig. 1h and k). The protein level of CCR2 in the DRG was slightly enhanced (1.2-fold, *p* < 0.05) 2 days after CCI, and then no changes were observed (Fig. 1l). Significantly enhanced mRNA level of CCR2 was observed on 7th (1.9-fold, *p* < 0.05) after CCI and even stronger upregulation on day 14th (3.2-fold, *p* < 0.001) (Fig. 1i).

### The Influence of Single and Repeated Administration of RS504393 on Pain-Related Behavior on the 7th Day Post-CCI

**Fig. 2:**
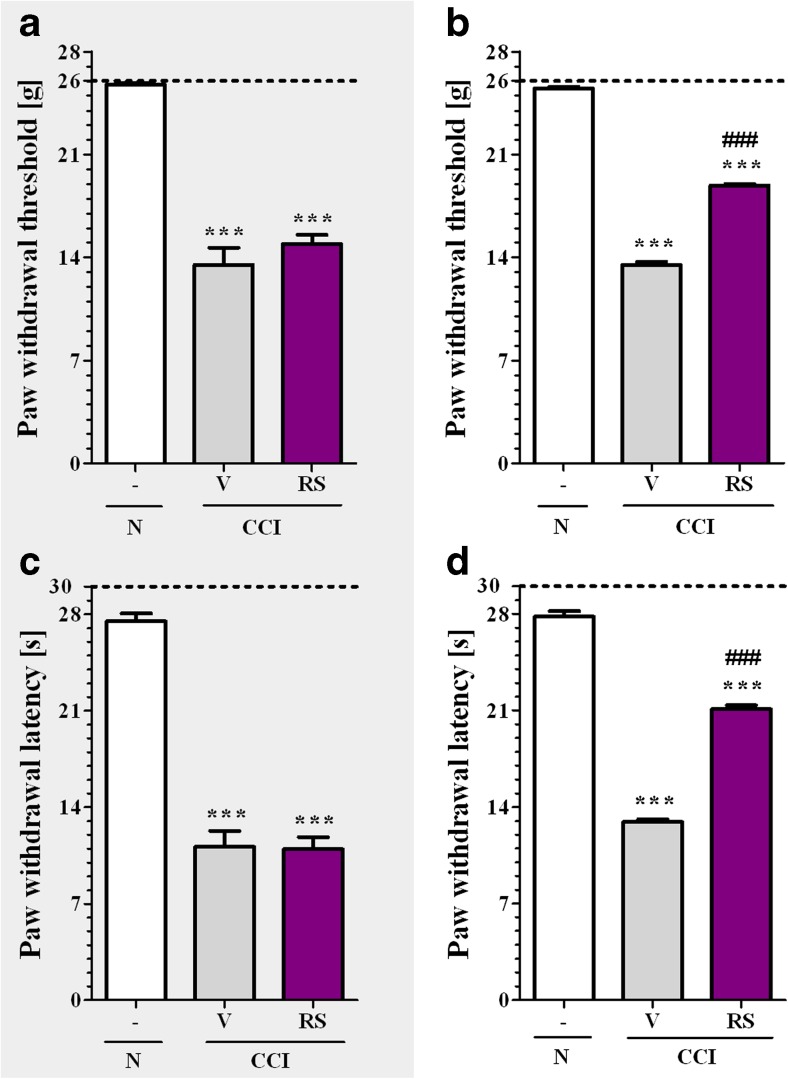
The influence of the single (5–8 rats per group; **a**, **c**) and repeated (14–20 rats per group; **b**, **d**) administration of RS504393 (RS; 20 μg/5 μl; *i.t*.; 16 h and 1 h before CCI and then once a day for 7 days) on pain-related behavior (**a**, **b** von Frey test; **c**, **d**, cold plate test) on the 7th day post-CCI, 30 min after RS or V injection. Tactile and thermal hypersensitivity were assessed at 30 min and 35 min, respectively. The data are presented as the mean ± SEM. The horizontal dotted line shows the cut-off value. The intergroup differences were analyzed using ANOVA with Bonferroni”s multiple comparisons test. ^***^ *p* < 0.001 indicates differences between naive rats and V-treated/RS-treated CCI-exposed rats; ^###^ *p* < 0.001 indicates differences vs. V-treated CCI-exposed rats. *Abbreviations; CCI, chronic constriction injury; N, naive; RS, RS504393; V, vehicle*

In the von Frey test the paw ipsilateral to the injury significantly responded to a stimulation in vehicle-treated CCI-exposed rats (*p* < 0.001, Fig. 2a, b). Single injection of RS504393 did not influence the level of pain-related behavior induced by mechanical stimulus in CCI-exposed rats (Fig. 2a). In contrast, pretreatment and repeated administration of RS504393 significantly (*p* < 0.001) attenuated CCI-induced tactile hypersensitivity 30 min after last injection (Fig. 2b).

In the cold plate test the paw ipsilateral to the injury significantly responded to a stimulation in vehicle-treated CCI-exposed rats (*p* < 0.001, Fig. 2c, d). Single injection of RS504393 did not influence the level of pain-related behavior induced by thermal stimulus in CCI-exposed rats (Fig. 2c). In contrast, pretreatment and repeated administration of RS504393 significantly (*p* < 0.001) attenuated CCI-induced thermal hypersensitivity 30 min after last injection (Fig. 2d).

### The Influence of RS504393 on Iba-1, CD4 and CD8 Protein Levels in the Spinal Cord and DRG 7 days after CCI

**Fig. 3:**
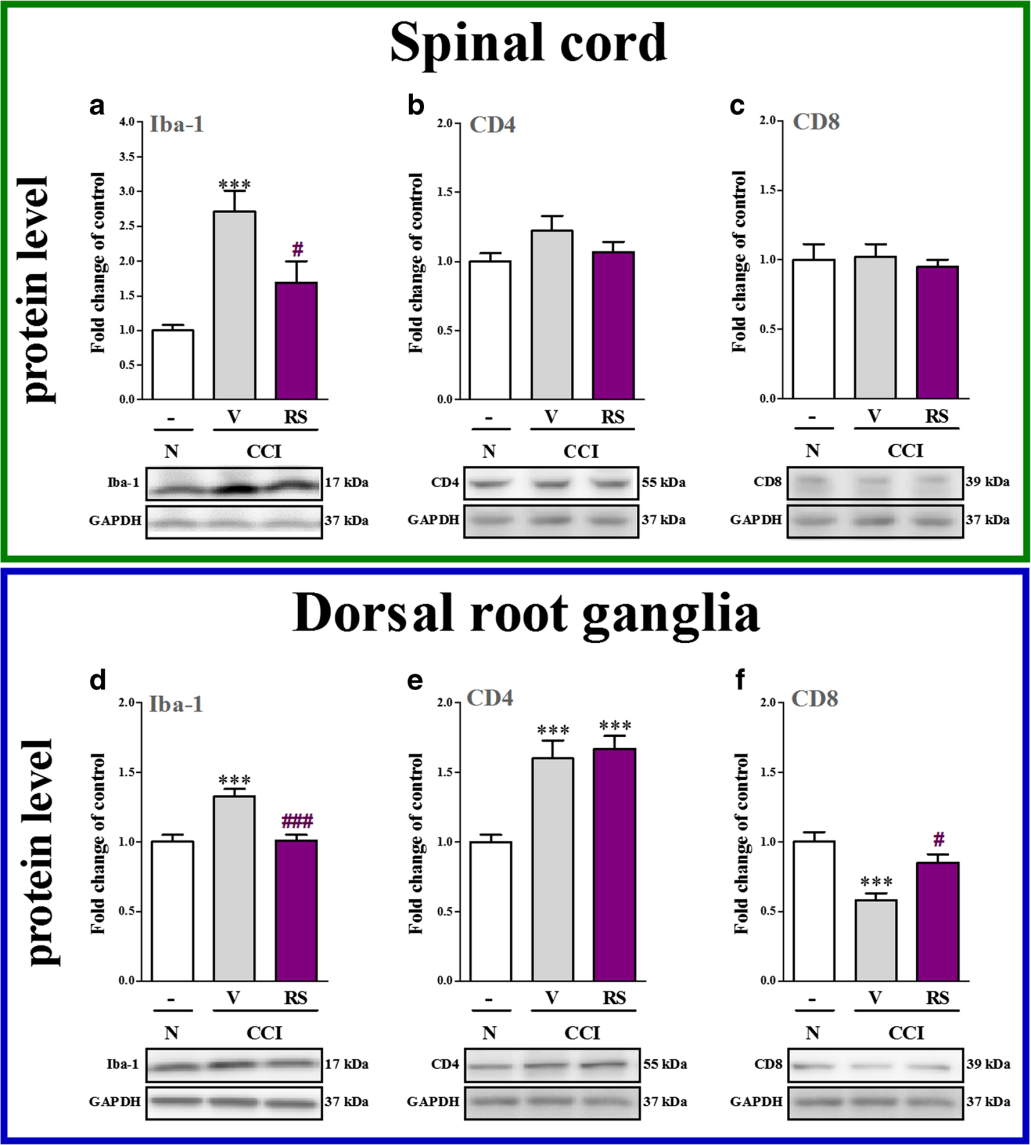
The influence of the repeated administration of RS504393 (RS; 20 μg/5 μl; *i.t*.; 16 h and 1 h before CCI and then once a day for 7 days) on protein (**a**–**f**) levels of Iba-1 (**a**, **d**), CD4 (**b**, **e**), CD8 (**c**, **f**) in the spinal cord and DRG on the 7th day after CCI. The data are presented as the mean fold changes of control ± SEM (5–9 samples per group). The intergroup differences were analyzed using ANOVA with Bonferroni’s multiple comparisons test. ^***^ *p* < 0.001 indicate differences between naive rats and V-treated/RS-treated CCI-exposed rats. ^#^ *p* < 0.05 and ^###^ *p* < 0.001 indicate differences vs. V-treated CCI-exposed rats. *Abbreviations: CCI, chronic constriction injury; N, naive; V, vehicle; RS, RS504393*

Iba-1 protein level was upregulated 2.7-fold (*p* < 0.001) in the spinal cord in CCI-exposed rats compared with naive rats (Fig. 3a). RS504393 decreased 1.6-fold the spinal protein level of Iba-1 (*p* < 0.05, Fig. 3a). No changes were observed in the level of CD4 in vehicle-treated and RS504393-treated CCI-exposed rats (Fig. 3b). Similarly, the protein level of CD8 also remains unchanged in the spinal cord (Fig. 3c).

In the DRG, the protein level of Iba-1 was upregulated 1.3-fold (*p* < 0.001), in the vehicle-treated CCI-exposed rats compared with naive rats (Fig. 3d). RS504393 significantly decreased (1.3-fold, *p* < 0.001) the spinal level of Iba-1 (Fig. 3d). The protein level of CD4 was upregulated in the DRG 1.6-fold (*p* < 0.001) in the CCI-exposed rats compared with naive rats (Fig. 3e) and RS504393 treatment did not influence these changes (Fig. 3e). CD8 protein level was downregulated 1.7-fold (*p* < 0.001) in the DRG in the CCI-exposed rats compared with naive rats; however RS504393 treatment prevented these changes (*p* < 0.05; Fig. 3f).

### The Influence of RS504393 on IL-1alpha, IL-1beta, IL-1RA and IL-1R1 mRNA and Protein Levels in the Spinal Cord and DRG 7 days after CCI

**Fig. 4:**
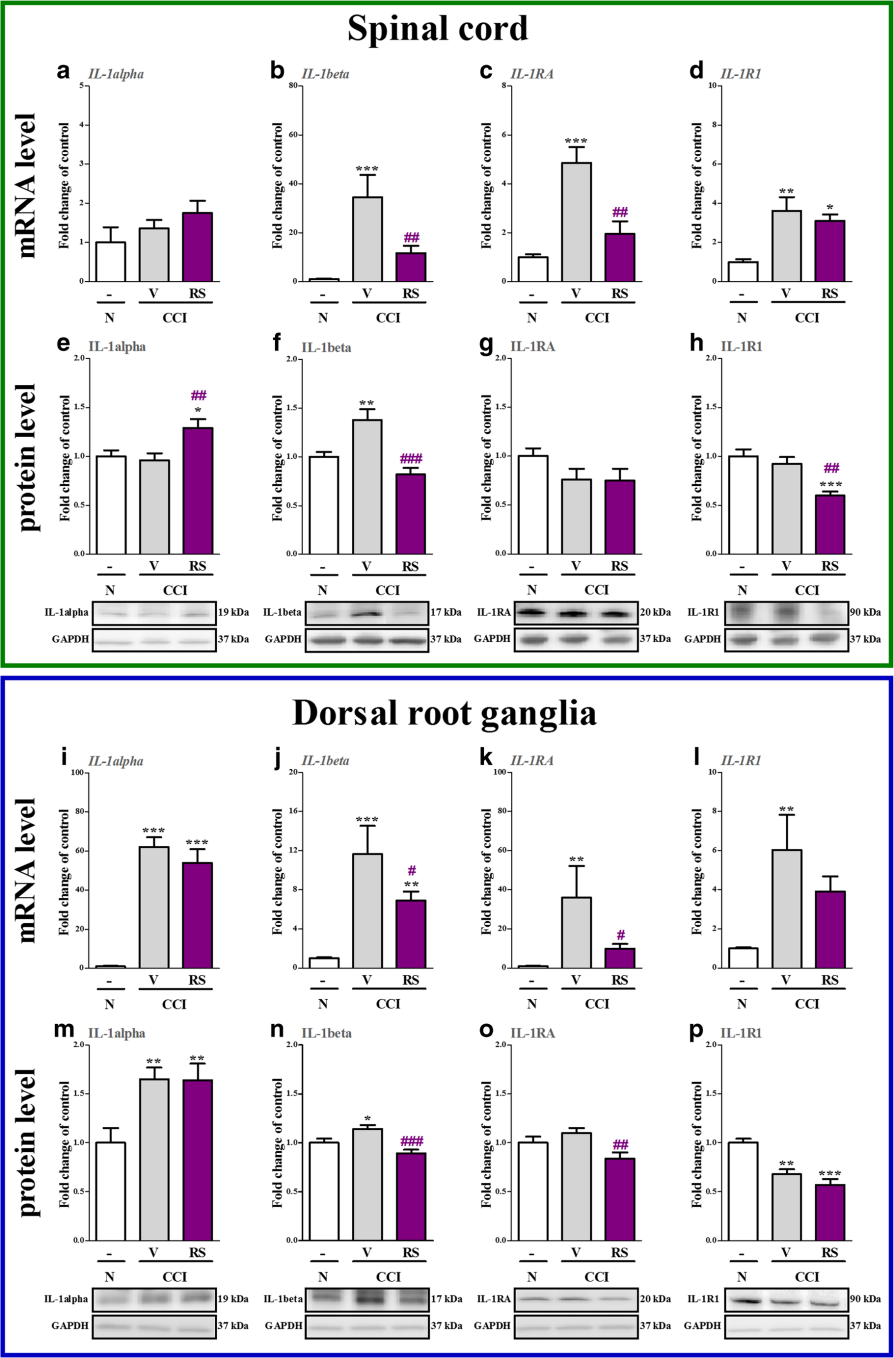
The influence of the repeated administration of RS504393 (RS; 20 μg/5 μl; *i.t*.; 16 h and 1 h before CCI and then once a day for 7 days) on mRNA (**a**–**d**, **i**–**l**) and protein (**e**–**h**, **m**–**p**) levels of IL-1alpha (**a**, **e**, **i**, **m**), IL-1beta (**b**, **f**, **j**, **n**), IL-1RA (**c**, **g**, **k**, **o**) and IL-1R1 (**d**, **h**, **l**, **p**) in the spinal cord and DRG on the 7th day after CCI. The data are presented as the mean fold changes of control ± SEM of 4–7 and 5–9 samples per group in RT-qPCR and Western blot analysis, respectively. The intergroup differences were analyzed using ANOVA with Bonferroni’s multiple comparisons test. ^*^ *p* < 0.05, ^**^ *p* < 0.01 and ^***^ *p* < 0.001 indicate differences between naive rats and V-treated/RS-treated CCI-exposed rats. ^#^ *p* < 0.05, ^##^ *p* < 0.01 and ^###^ *p* < 0.001 indicate differences vs. V-treated CCI-exposed rats. *Abbreviations: CCI, chronic constriction injury; N, naive; V, vehicle; RS, RS504393*

In the spinal cord, no changes were observed in the mRNA and protein levels of IL-1alpha (Fig. 4a and e) in vehicle-treated CCI-exposed rats compared with naive rats. RS504393 increased 1.3-fold (*p* < 0.01) the protein level of IL-1alpha (Fig. 4e); however, the mRNA level remained unchanged (Fig. 4a) in the spinal cord. IL-1beta mRNA and protein levels were upregulated 34.4-fold (*p* < 0.001) and 1.4-fold (*p* < 0.01), respectively in the spinal cord (Fig. 4b and f) in the CCI-exposed rats compared with naive rats. RS504393 decreased 2.9-fold (*p* < 0.01) the mRNA and 1.75-fold (*p* < 0.001) protein spinal levels of IL-1beta (Fig. 4b and f, respectively). IL-1RA mRNA level was increased 4.85-fold (*p* < 0.001, Fig. 4c) in the spinal cord; however, no changes were observed in the protein level (Fig. 4g) in the CCI-exposed rats compared with naive rats. RS504393 decreased 2.4-fold (*p* < 0.01) the spinal mRNA level of IL-1RA (Fig. 4c) but did not influence the protein level (Fig. 4g). IL-1R1 mRNA level was increased 3.6-fold (*p* < 0.01, Fig. 4d) in the spinal cord in the CCI-exposed rats compared with naive rats, but no changes were observed in the protein level (Fig. 4h). Although RS504393 did not influence the mRNA level (Fig. 4d), it decreased the protein level of IL-1R1 1.5-fold (*p* < 0.01) in the spinal cord (Fig. 4h).

In the DRG, the mRNA and protein levels of IL-1alpha were upregulated 62.1-fold (*p* < 0.001) and 1.65-fold (*p* < 0.001), respectively (Fig. 4i and m), in the vehicle-treated CCI-exposed rats compared with naive rats. RS504393 did not influence the mRNA and protein levels of IL-1alpha in the DRG (Fig. 4i and m, respectively). IL-1beta mRNA and protein levels were upregulated in the DRG 11.6-fold (*p* < 0.001) and 1.1-fold (*p* < 0.05), respectively (Fig. 4j and n), in the CCI-exposed rats compared with naive rats. RS504393 reduced the mRNA (1.6-fold, *p* < 0.05) and protein (1.2-fold, *p* < 0.001) levels of IL-1beta in the DRG (Fig. 4j and n, respectively). IL-1RA mRNA level was increased 36-fold (*p* < 0.001, Fig. 4k) in the DRG in the CCI-exposed rats compared with naive rats, although no changes were observed in the protein level (Fig. 4o). RS504393 downregulated the mRNA (3.6-fold, *p* < 0.05) and protein (1.3-fold, *p* < 0.001) levels of IL-1RA in the DRG (Fig. 4k and o, respectively). IL-1R1 mRNA level was increased 6-fold *p* < 0.001 Fig. 4l) in the CCI-exposed rats compared with naive rats, whereas the protein level of IL-1R1 was decreased 1.4-fold (*p* < 0.001, Fig. 4p) in the DRG. RS504393 did not influence the level of IL-1R1 (Fig. 4l and p).

### The Influence of RS504393 on IL-18, IL-18BP and IL-18R mRNA and Protein Levels in the Spinal Cord and DRG 7 days after CCI

**Fig. 5:**
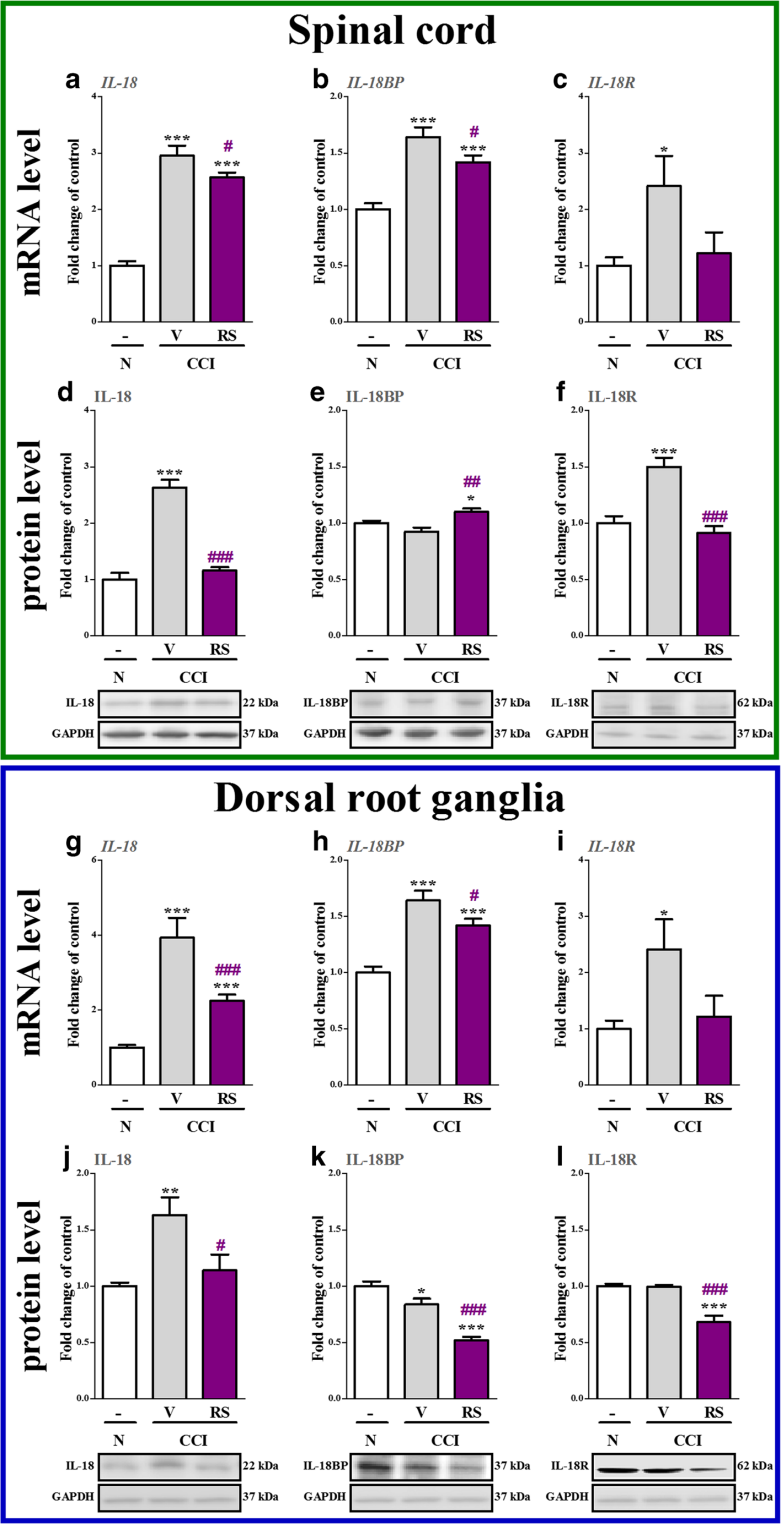
The influence of the repeated administration of RS504393 (RS; 20 μg/5 μl; *i.t*.; 16 h and 1 h before CCI and then once a day for 7 days) on mRNA (**a**–**c**, **g**–**i**) and protein (**d**–**f**, **j**–**l**) levels of IL-18 (**a**, **d**, **g**, **j**), IL-18BP (**b**, **e**, **h**, **k**) and IL-18R (**c**, **f**, **i**, **l**) in the spinal cord and dorsal root ganglia on the 7th day after CCI. The data are presented as the mean fold changes of control ± SEM of 4–7 and 5–9 samples per group in RT-qPCR and Western blot analysis, respectively. The intergroup differences were analyzed using ANOVA with Bonferroni’s multiple comparisons test. ^*^ *p* < 0.05, ^**^ *p* < 0.01 and ^***^ *p* < 0.001 indicate differences between naive rats and V-treated/RS-treated CCI-exposed rats. ^#^ *p* < 0.05, ^##^ *p* < 0.01 and ^###^ *p* < 0.001 indicate differences vs. V-treated CCI-exposed rats. *Abbreviations: CCI, chronic constriction injury; N, naive; V, vehicle; RS, RS504393*

IL-18 mRNA and protein levels were upregulated (*p* < 0.001) in the spinal cord 2.95-fold (Fig. 5a) and 2.6-fold (Fig. 5d), respectively, in the CCI-exposed rats compared with naive rats. RS504393 reduced the mRNA 1.1-fold (*p* < 0.05) and protein 2.1-fold (*p* < 0.001) levels of IL-18 in the spinal cord (Fig. 5a and d, respectively). IL-18BP mRNA level was increased 1.6-fold (*p* < 0.001, Fig. 5b) in the CCI-exposed rats compared with naive rats, but no changes were observed in the protein level of IL-18BP (Fig. 5e). RS504393 decreased the mRNA 1.1-fold (*p* < 0.05) and increased the protein 1.2-fold (*p* < 0.01) spinal levels of IL-18BP (Fig. 5b and E). IL-18R mRNA and protein levels were upregulated in the spinal cord 2.4-fold (*p* < 0.05, Fig. 5c) and 1.5-fold (*p* < 0.001, Fig. 5f) in the CCI-exposed rats compared with naive rats. Although RS504393 did not influence the mRNA IL-18R level (Fig. 5c), whereas it decreased the protein level 1.6-fold (*p* < 0.001, Fig. 5f).

In the DRG, IL-18 mRNA and protein levels were upregulated 3.9-fold (*p* < 0.001, Fig. 5g) and 1.6-fold (*p* < 0.01, Fig. 5j), respectively, in the CCI-exposed rats compared with naive rats. RS504393 reduced the mRNA 1.7-fold (*p* < 0.001) and protein 1.45-fold (*p* < 0.05) levels of IL-18 in the DRG (Fig. 5g and j, respectively). IL-18BP mRNA level was increased 1.9-fold (*p* < 0.001, Fig. 5h) in the CCI-exposed rats compared with naive rats, but the protein level of IL-18BP was decreased 1.2-fold (*p* < 0.05, Fig. 5k). RS504393 reduced the mRNA 1.5-fold (*p* < 0.05) and protein 1.6-fold (*p* < 0.001) levels of IL-18BP in the DRG (Fig. 5h and k). IL-18R mRNA level was upregulated 2.2-fold (*p* < 0.05, Fig. 5i) in the DRG, whereas no changes were observed in the protein level (Fig. 5l) in the CCI-exposed rats compared with naive rats. Although RS504393 did not influence the mRNA IL-18R level (Fig. 5i) in the DRG, but it decreased the protein level 1.4-fold (*p* < 0.001, Fig. 5l).

### The Influence of RS504393 on IL-6, IL-10 and iNOS mRNA and Protein Levels in the Spinal Cord and DRG 7 days after CCI

**Fig. 6:**
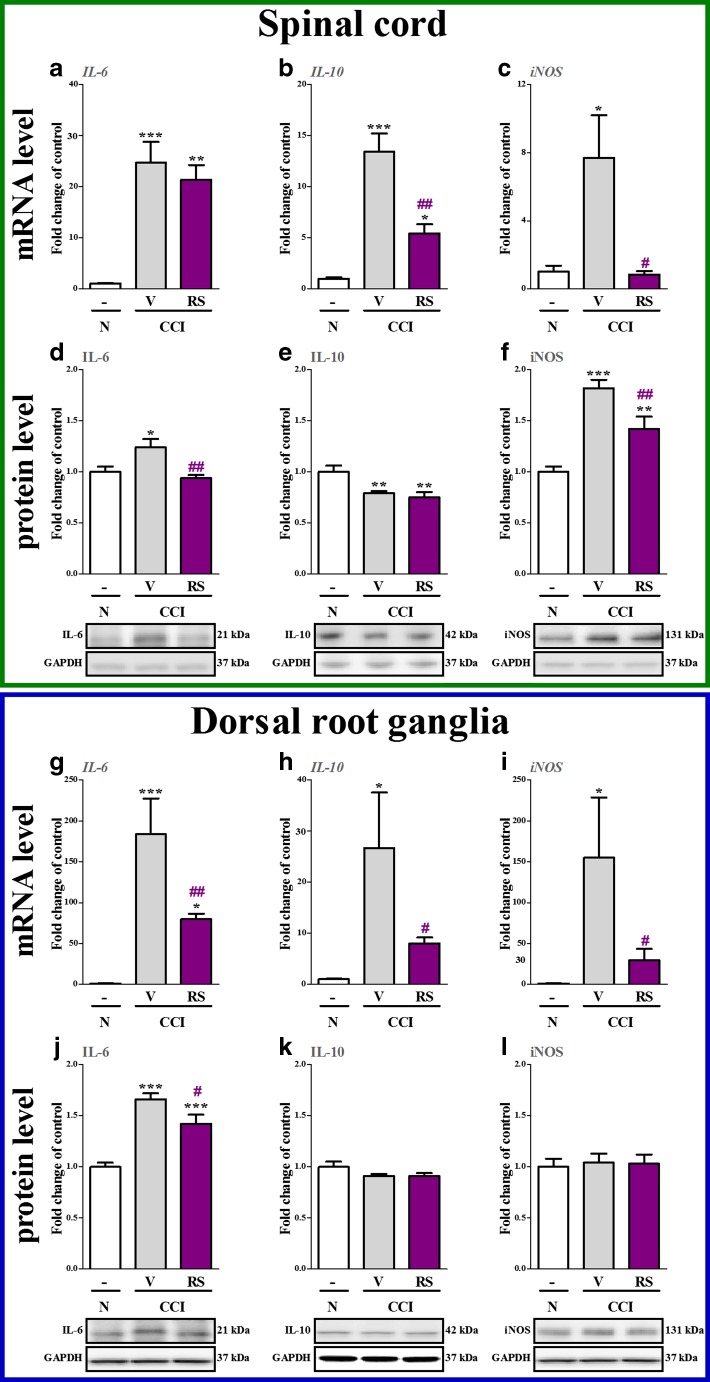
The influence of the repeated administration of RS504393 (RS; 20 μg/5 μl; *i.t*.; 16 h and 1 h before CCI and then once a day for 7 days) on mRNA (**a**–**c**, **g**–**i**) and protein (**d**–**f**, **j**–**l**) levels of IL-6 (**a**, **d**, **g**, **j**), IL-10 (**b**, **e**, **h**, **k**) and iNOS (**c**, **f**, **i**, **l**) in the spinal cord and DRG on the 7th day after CCI. The data are presented as the mean fold changes of control ± SEM of 4–7 and 5–9 samples per group in RT-qPCR and Western blot analysis, respectively. The intergroup differences were analyzed using ANOVA with Bonferroni’s multiple comparisons test. ^*^ *p* < 0.05, ^**^ *p* < 0.01 and ^***^ *p* < 0.001 indicate differences between naive rats and V-treated/RS-treated CCI-exposed rats. ^#^ *p* < 0.05 and ^##^ *p* < 0.01 indicate differences vs. V-treated CCI-exposed rats. *Abbreviations: CCI, chronic constriction injury; N, naive; V, vehicle; RS, RS504393*

IL-6 mRNA and protein levels were upregulated in the spinal cord 24.7-fold (*p* < 0.001, Fig. 6a) and 1.2-fold (*p* < 0.05, Fig. 6d), respectively, in the CCI-exposed rats compared with naive rats. RS504393 diminished the protein level of IL-6 1.3-fold (*p* < 0.01, Fig. 6d) and did not influence the mRNA level (Fig. 6a). IL-10 mRNA level was upregulated 13.4-fold (*p* < 0.001, Fig. 6b) in the CCI-exposed rats compared with naive rats; however, the protein level was downregulated 1.2-fold (*p* < 0.01, Fig. 6e). RS504393 decreased the mRNA 2.48-fold (*p* < 0.01) and did not influence the protein level of IL-10 at the spinal cord level (Fig. 6b and e). The mRNA and protein levels of iNOS were upregulated 7.7-fold (*p* < 0.05, Fig. 6c) and 1.8-fold (*p* < 0.001, Fig. 6f), respectively, at the spinal cord level in the CCI-exposed rats compared with naive rats. RS504393 decreased the mRNA (9.6-fold, *p* < 0.05) and protein levels (1.3-fold, *p* < 0.01) of iNOS in the spinal cord (Fig. 6c and f, respectively).

In the DRG, IL-6 mRNA and protein level was upregulated 183.6-fold (*p* < 0.001, Fig. 6g) and 1.7-fold (*p* < 0.001, Fig. 6j), respectively, in the CCI-exposed rats compared with naive rats. RS504393 diminished the mRNA (2.3-fold, *p* < 0.01) and protein (1.21-fold, *p* < 0.05) levels of IL-6 (Fig. 6g and j, respectively). IL-10 mRNA level was upregulated 26.7-fold (*p* < 0.05, Fig. 6h) in the CCI-exposed rats compared with naive rats, but no changes were observed in the protein level (Fig. 6k). RS504393 decreased the mRNA (3.3-fold, *p* < 0.05, Fig. 6h) and did not influence the protein level of IL-10 in the DRG (Fig. 6k). The mRNA level of iNOS in the DRG was upregulated 154.9-fold (*p* < 0.001, Fig. 6i), although no changes were observed in the protein level (Fig. 6l) in the CCI-exposed rats compared with naive rats. RS504393 decreased the mRNA (5.3-fold, *p* < 0.05) and did not influence the protein level of iNOS in the DRG (Fig. 6i and l, respectively).

### The Influence of the Repeated Administration of RS504393 on Opioid Effectiveness on the 7th Day Post-CCI

**Fig. 7:**
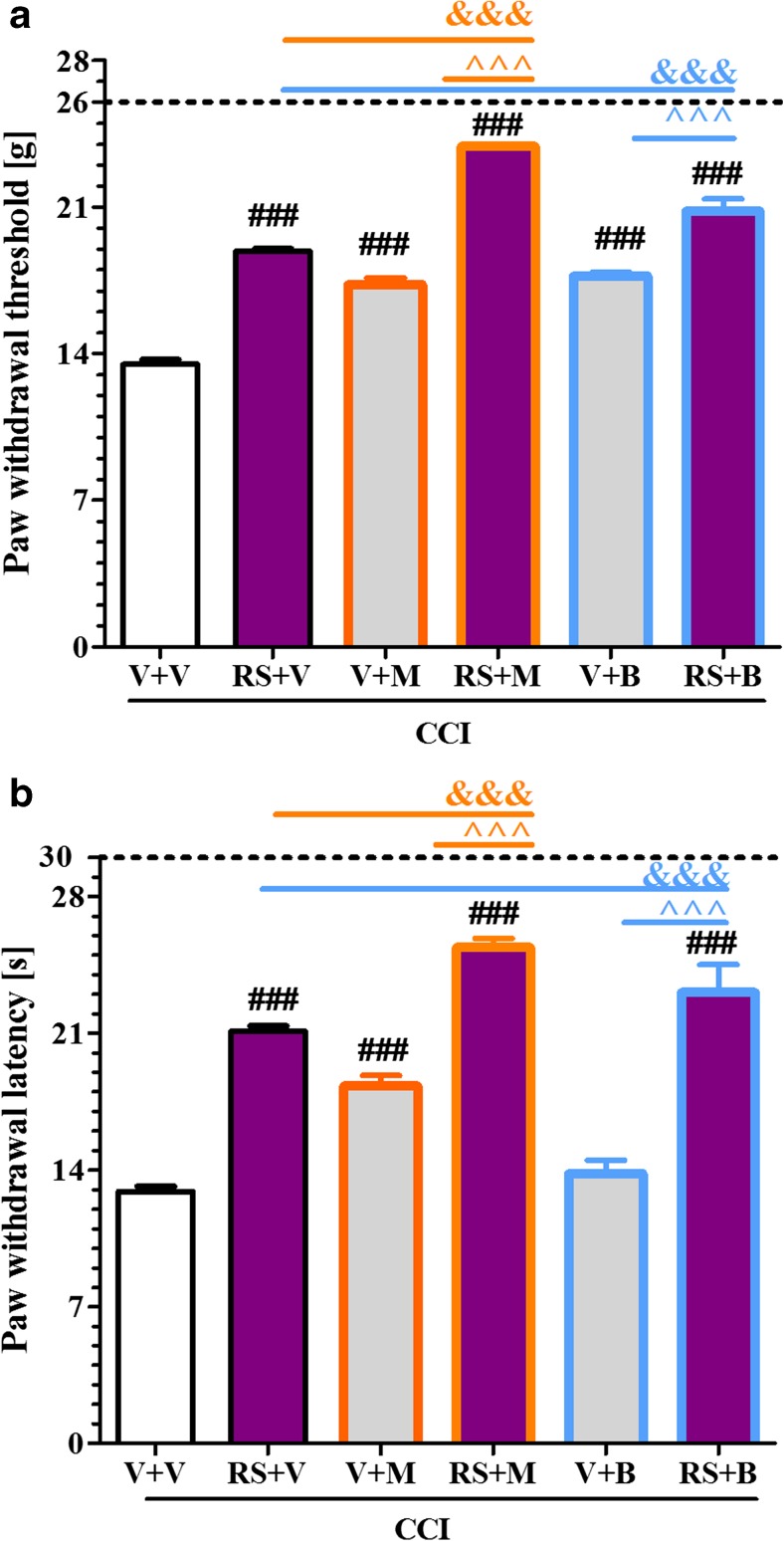
The influence of the repeated administration of RS504393 (RS; 20 μg/5 μl; *i.t*.; 16 h and 1 h before CCI and then once a day for 7 days) on pain-related behavior (A, von Frey test; B, cold plate test) and the analgesic effects of morphine (M; 2.5 μg/5 μl; single dose *i.t.*; on the 7th day post-CCI, 60 min after RS or V injection) and buprenorphine (B; 2.5 μg/5 μl; single dose *i.t.*; on the 7th day post-CCI, 60 min after RS or V injection) in CCI-exposed rats. Tactile and thermal hypersensitivity were assessed at 30 min and 35 min, respectively, after the last RS504393 injection and once again after single morphine or buprenorphine injections. The data are presented as the mean ± SEM of 5–14 rats per group. The horizontal dotted line shows the cut-off value. The intergroup differences were analyzed using ANOVA with Bonferroni”s multiple comparisons test. ^###^ *p* < 0.001 indicates differences vs. V + V-treated CCI-exposed rats; ^&&&^ *p* < 0.001 indicates differences vs. RS + V-treated CCI-exposed rats; ^^^*p* < 0.001 indicates differences between V + M- or V + B-treated vs. RS + M- or RS + B-treated, CCI-exposed rats. *Abbreviations: B, buprenorphine; CCI, chronic constriction injury; M, morphine; N, naive; RS, RS504393; V, vehicle*

In von Frey test the single injections of the respective opioids induced similar analgesic effects as RS504393. The combined administration of RS504393 and morphine or buprenorphine resulted in substantially more effective analgesia (*p* < 0.001, Fig. 7a). In cold plate test the single injections of respective opioids induced lower analgesic effects than the CCR2 antagonist. The combined administration of RS504393 and morphine or buprenorphine resulted in substantially enhanced analgesic effects (*p* < 0.001, Fig. 7b).

## Discussion

It has been previously demonstrated that in the CCI model a strong tactile and thermal hypersensitivity developed on day 2 and lasted until day 14 after sciatic nerve injury (Pilat et al. 2016; Jurga et al. 2016). Similar time course is observed in the microglial cells activation, with the strongest upregulation of Iba-1 on day 7 post-CCI (Pilat et al. 2016; Jurga et al. 2016), which we also confirmed in our current study. Additionally, we revealed simultaneous upregulation of spinal protein level of CCL2 and CCR2 on 2nd and 7th day after CCI. In DRG the strongest upregulation of protein level of both, CCL2 and CCR2 was observed on 2nd day post-CCI and in case of CCL2 lasted until day 14th.

Our behavioral experiments demonstrated that repeated, but not single, intrathecal administration of RS504393 not only attenuated tactile and thermal hypersensitivity but also enhanced analgesic properties of morphine and buprenorphine. These results complemented our previous findings describing the analgesic effects of RS504393 and its beneficial influence on CCI-elevated spinal upregulation of CCR2 and CCL2 under neuropathy (Piotrowska et al. 2016a). Our results are in agreement with others, showing that single intracisternal injections of CCR2 antagonist reduced neuropathic pain induced by inferior alveolar nerve transection (Zhang et al. 2012) and lumbar disc herniation (Zhu et al. 2014). Importantly, our study is, to our knowledge, the first to identify that chronic administration of RS504393 significantly enhanced opioid effectiveness in a rat neuropathic pain model. We hypothesize that the beneficial properties of RS504393 resulted from antagonizing CCR2, as it has been shown in our previous paper (Piotrowska et al. 2016a) and also in other publications (Berkhout et al. 2003; Pevida et al. 2012; Zhang et al. 2012). Nevertheless, Mirzadegan et al. (2000) demonstrated that RS504393 has also high affinity for α1a adrenergic receptor, which is known to be involved in the inflammatory response occurring after peripheral nerve injury (Drummond 2014) and important for generating and transmitting neuropathic pain (Cheng et al. 2014). This might be relevant for the analgesic effects which we observed, however it needs to be evaluated in the future.

The CCR2 is expressed by spinal microglia, astrocytes and neurons, and CCL2 preferentially binds this receptor (Kurihara and Bravo 1996; Jung et al. 2009; Gao and Ji 2010). The nociception can be increased directly by the activation of CCR2 which is located on neurons. It was previously showed that the intrathecal injection of CCL2 induced hyperalgesia and ERK activation in superficial dorsal horn neurons (Gao et al. 2009). Huang et al. (2014) showed that CCL2 injected intrathecally induced the release of TNF-α, which subsequently augmented an excitatory glutamatergic transmission in substantia gelatinosa neurons. Moreover, studies with the patch clamp technique revealed that CCL2 increased excitatory synaptic transmission as well as both AMPA- and NMDA-induced currents (Gao et al. 2009). Recently, Jin et al. (2015) showed that CCL2 was also significantly involved in the activation of spinal microglia associated with pain development. The intrathecal administration of CCL2 neutralizing antibody reduced the expression of microglial marker OX-42, and thus pain behavior (Jin et al. 2015). We observed similar effects in our previous studies, where we used the CCR2 antagonist (Piotrowska et al. 2016a). Our new data suggest that on day 7 after the CCI, the level of CCL2 increased in parallel with the upregulation of Iba-1 and CD4-positive cells marker, and downregulation of CD8-positive cells marker. Therefore, it seems that the CCR2 activation increases nociception through both direct and indirect manner.

Several studies have suggested that endogenous CCL2 is one of the key mediators of spinal glia activation after nerve injury (Thacker et al. 2009; Gao and Ji 2010; Parpura and Zorec 2010; Zhao et al. 2012; Zhu et al. 2014). Baamonde et al. (2011) showed that intrathecal administration of CCL2 provokes glial activation and the release of pronociceptive molecules, such as IL-1beta, that together with CCL2, leads to the sensitization of NMDA and AMPA receptors and contributes to the development of thermal hypersensitivity. In 2016, Yang et al., suggested that CCL2 can promote microglia cytokine secretion after nerve injury and that CCL2 RNAi inhibited the expression of IL-1beta, IL-6 and tumor necrosis factor alpha (TNF-α) by spinal microglia (Yang et al. 2016). These findings are in agreement with our recently published results showing that microglia inhibitor minocycline decreased CCI-induced pain and, in parallel, reduced spinal CCL2/CCR2 signaling (Piotrowska et al. 2016a). We have also reported that RS504393 prevented CCI-induced spinal activation of microglia in a neuropathic pain model (Piotrowska et al. 2016a). Here, we provide the first evidence that a direct blockade of CCL2/CCR2 signaling modulates the level of some nociceptive factors in a rat model of neuropathic pain.

IL-1 family consists of numerous cytokines, sometimes with opposing roles in nociceptive transmission under neuropathy. Our current study indicated that the CCI-induced development of tactile and thermal hypersensitivity is associated with the upregulation of IL-1beta in the spinal cord and DRG, which is in agreement with previous reports (Pilat et al. 2015). Additionally, we demonstrated for the first time that RS504393 prevented the CCI-elevated upregulation of pronociceptive IL-1beta in both examined structures, suggesting that this can be one of the reasons of its strong analgesic properties. These findings are in line with a previous study that showed that RS504393 treatment significantly reduced the lipopolysaccharide (LPS)-elevated level of IL-1beta in mice with acute lung injury (Yang et al. 2010). Interestingly, we also observed increased spinal protein level of antinociceptive IL-1alpha after chronic injections of RS504393, which likely contributes to the beneficial effects of the CCR2 antagonist. Different situation is observed in the DRG, where strong upregulation of antinociceptive IL-1alpha induced by CCI was not changed by RS504393. The protein level of antinociceptive IL-1RA was not changed in both examined structures. However surprisingly, in the DRG it was slightly decreased after RS504393 treatment, although the exact mechanism underlying this phenomenon remains unclear and requires further investigation. The aforementioned interleukins act by binding to the functionally active IL-1R1 (Obreja et al. 2002). We observed that the CCI induced no changes in IL-1R1 in the spinal cord and only slightly decreased its level in the DRG. After the nerve injury the presence of IL-1R1 on sensory neurons suggests the possibility that IL-1beta might directly influence nociceptive transmission (Oka et al. 1994; Obreja et al. 2002), therefore IL-1RA successfully decreases neuropathic pain (Pilat et al. 2015). We assume that IL-1R1 is functionally active and that its turnover might be enhanced in pathology. Thus, we did not observe higher protein levels for this receptor, which is crucial for pronociceptive IL-1beta. Our results are in agreement with those obtained by Nadeau et al. (2011). In 1999, Sommer et al., showed that epineural injections of anti-IL-1R1 antibodies into the spinal cord reduced both pain-related behavior in mice following CCI (Sommer et al. 1999). Here, we demonstrated that the CCR2 antagonist decreased the spinal protein level of IL-1R1, which is crucial for impaired nociceptive transmission under neuropathic pain.

IL-18 is another pleiotropic pronociceptive cytokine that belongs to IL-1 superfamily (Pilat et al. 2016; Samarani et al. 2016). The pro-inflammatory effects of IL-18 are tightly controlled by its naturally occurring antagonist, called IL-18BP, which is produced due to a negative feedback mechanism (Dinarello and Fantuzzi 2003; Samarani et al. 2016). Under physiological conditions, most IL-18 is bound with IL-18BP and inactivated. Several studies demonstrated that the development of neuropathic pain after peripheral nerve injury is associated with spinal upregulation of mRNA and the protein level of IL-18 and its specific receptor, IL-18R (Miyoshi et al. 2008; Rojewska et al. 2014b; Popiolek-Barczyk et al. 2015; Pilat et al. 2016). These findings are in line with the results obtained in the current experiment. We observed CCI-induced upregulation of IL-18 mRNA and protein levels in the spinal cord and DRG. Additionally, we provide the first evidence that blocking CCR2 by RS504393 significantly prevented enhanced CCI-elevated upregulation of pronociceptive IL-18 in both examined structures, which may be crucial for its analgesic properties. Moreover, we showed that the CCR2 antagonist decreased the protein level of IL-18R in the spinal cord and DRG, which can be beneficial because the pronociceptive IL-18 loses its main target. The injection of IL-18BP results in strong antiallodynic and antihyperalgesic effects in nerve-injured rats (Miyoshi et al. 2008; Pilat et al. 2016). The CCR2 antagonist significantly increased the spinal protein level of antinociceptive IL-18BP, which highly probable to exert a positive impact on nociceptive transmission. However, the protein level of IL-18BP in the DRG in RS504393-treated rats was even more decreased than in the vehicle-treated group, which remains unclear. All these results suggest that IL-18/IL-18R pathway is involved in neuropathic pain development to a greater extent at the spinal cord level compared to DRG.

Several studies have indicated that IL-6, which is secreted mostly by macrophages and activated microglia, is strongly increased after peripheral nerve injury. Therefore, it was suggested as one of the first factors to play a key role in neuropathic pain development (DeLeo et al. 1996; Mika et al. 2008; Lee et al. 2010). Here, we confirmed the strongly enhanced level of IL-6 in the spinal cord and DRG in CCI-exposed rats. It was previously reported that administration of IL-6 neutralizing antibodies reduced pain behavior in neuropathic pain models (Schoeniger-Skinner et al. 2007; Mika et al. 2008; Lee et al. 2010). Yang et al. (2010) showed in an acute lung injury model that the protein level of IL-6 in the bronchoalveolar lavage fluid after LPS challenge was significantly lower in CCR2 knockout mice compared to C57BI/6 J mice. Moreover, Nanki et al. (2001) suggested that CCL2 enhances extracellular signal-regulated protein kinases 1 and 2 (ERK1/2) activation due to CCR2 and, therefore, induces IL-6 production by fibroblast-like synoviocytes in rheumatoid arthritis. We demonstrated that RS504393 significantly reduced the protein level of IL-6 in both examined structures, which might be one of the reasons behind its beneficial properties.

The inducible nitric oxide synthase (iNOS) is a pronociceptive factor that is expressed by both, microglia and astrocytes (Tran et al. 1997; Possel et al. 2000), however in the early phase of the neuropathic pain the main source of iNOS are highly activated microglial cells (Popiolek-Barczyk et al. 2015). Here, we observed the enhanced protein level of iNOS in the spinal cord, but not in DRG in CCI-exposed rats. Previously, we reported that minocycline, a microglial inhibitor, reduced the CCI-elevated protein level of iNOS (Makuch et al. 2013). Importantly, in our current study, we also demonstrated that RS504393 significantly decreased the level of iNOS at the spinal cord level, which might be a consequence of its inhibition of microglial cell activation, as we recently reported (Piotrowska et al. 2016a).

IL-10 is an example of an antinociceptive cytokine, which exhibits increased mRNA levels after sciatic nerve injury, as previously described (Mika et al. 2008; Rojewska et al. 2014b). These findings are in line with our current results, in which we observed increased mRNA levels of IL-10 in the spinal cord and DRG. It was suggested that IL-10 may interact with microglia through an inhibition of pronociceptive cytokine release, e.g., IL-1beta, IL-6, and TNF-alpha (Relton and Rothwell 1992; Sawada et al. 1999). However, RS504393 decreased the mRNA level of IL-10 but did not influence the protein level in both examined structures.

Opioids are widely used in treatment of chronic pain. Nevertheless, in the neuropathic pain, opioids exhibit lower effectiveness than in other pain conditions (Watkins et al. 2005; Szczudlik et al. 2014). The mechanism underlying this phenomenon is still poorly understood. Several studies reported that morphine analgesic properties are influenced by profoundly activated microglial cells that release pronociceptive factors during neuropathic pain (Ledeboer et al. 2005; Mika et al. 2007, 2009; Rojewska et al. 2014a, b; Zhang et al. 2015). It was demonstrated that chronic administration of minocycline delayed the development of morphine tolerance due to a reduction in spinal cord expressions of IL-1beta and IL-18 (Mika et al. 2009; Rojewska et al. 2014b; Zhang et al. 2015). Thereby, it was postulated that cytokines may modulate opioid receptor activity, and the excessive production of interleukins, such as IL-1beta and IL-18, might be of key importance for decreasing the opioid analgesic efficacy observed in neuropathic pain therapy (Johnston et al. 2004; Pilat et al. 2015, 2016). Several studies have indicated that opioid signaling is connected not only with interleukins but also with chemokines. Zhao et al. (2012) showed that chronic administration of morphine increased spinal CCL2, and its neutralizing antibody reduced morphine-induced spinal microglial activation and morphine tolerance. Moreover, a selective agonist of the μ opioid receptor (DAMGO) enhanced the expressions of CCL2, CCL5 and CXCL10 in human peripheral blood mononuclear cells (Wetzel et al. 2000). The overexpression of CXCL10 due to activation of CXCR3 and G_i_ protein results in an attenuation of opioid-induced analgesia. However, blocking CXCL10 spinal function enhanced the morphine antinociceptive properties in rats with cancer-induced bone pain (Ye et al. 2014). Thus, increasing evidence points to cross-talk between opioid and chemokine receptors, which both belong to the G-protein coupling receptors (GPCRs) superfamily. The fundamental mechanism governing the function of GPCRs is desensitization. Several chemokines (e.g., CCL2, CCL5, CXCL12, and CX3CL1) are known to interfere with the analgesic effects induced by opioids due to heterologous desensitization (Szabo et al. 2002; Chen et al. 2007). Szabo et al. (2002) reported that desensitization of the μ opioid receptor by CCL5 and CXCL12 disrupted the balance between analgesia and algesia. Previously, we reported that a CCR5 antagonist (maraviroc) enhanced the analgesic potency of morphine and buprenorphine (Kwiatkowski et al. 2016), which was associated with the lower levels of pronociceptive interleukins (Piotrowska et al. 2016b). In the current study, we demonstrated that CCR2 antagonist (RS504393) also enhanced the analgesic potency of morphine and buprenorphine in neuropathic rats. These effects correspond well with the reduction in CCI-elevated levels of CCL2 (Piotrowska et al. 2016a) and pronociceptive interleukins, such as IL-1beta, IL-18 and IL-6. A probable mechanism underlying the beneficial properties of RS504393 is the inhibition of microglial cell activation (Piotrowska et al. 2016a), responsible for releasing pronociceptive factors, which negatively influence opioid-induced analgesia. In our opinion, the activated spinal microglia is a key factor in the development of neuropathic pain, as well as in the efficacy of different opioid analgesics. We previously showed (Mika et al. 2014; Popiolek-Barczyk et al. 2014) that microglial inhibitor, minocycline, enhances the effectiveness of selective MOP, KOP and NOP, but not DOP ligands under neuropathic pain conditions. In our experiment, RS504393 acts as an inhibitor of microglial activation, therefore, it remains in agreement with our previously published data that both morphine (ligand of MOP, DOP and KOP) and buprenorphine (ligand of MOP, DOP, KOP, NOP) effectiveness is enhanced in a CCI model. However, the explanation of this phenomenon requires further insightful research. Moreover, this lets us assume that the enhancement of their effectiveness is undoubtedly related to MOP and KOP receptors, as in case of buprenorphine also with NOP. Furthermore, we hypothesize that the CCR2 antagonist, by preventing CCI-elevated upregulation of CCL2 and CCR2, inhibited heterologous desensitization between opioid and chemokine receptors, although this needs to be studied in the future.

## Conclusion

We propose CCR2 as a promising target for the manipulation of neuropathic pain. The low effectiveness of opioids in neuropathic pain is associated with significant changes in the production of pro- and antinociceptive factors. In our current study, we observed that targeting CCR2 restores the analgesic activities of morphine and buprenorphine. Simultaneously, RS504393 prevented the CCI-induced upregulation of pronociceptive factors, such as IL-1beta, IL-18, IL-6 and iNOS, due to inhibition of microglial activation and increased expression of antinociceptive IL-1alpha. This supports the hypothesis that the pharmacological modulation of neuroimmunological interactions via CCR2 may represent a new strategy for effective polytherapy with opioids in patient suffering from neuropathic pain.
